# Microfilaruria of *Dirofilaria immitis* in a dog from Italy

**DOI:** 10.1007/s00436-024-08206-0

**Published:** 2024-04-22

**Authors:** Livia Perles, Floriana Gernone, Giuseppe Menga, Riccardo Taurino, Stefania Fornelli, Marianna Covino, Umberto Russo, Iuliana Ionascu, Domenico Otranto

**Affiliations:** 1 Department of Veterinary Medicine, University of Bari, Valenzano, Italy; 2 Veterinary Diagnostic Laboratory ACV Triggiano, Triggiano, Italy; 3 Veterinary Clinic Santa Chiara, Brindisi, Italy; 4 University of Agronomical Sciences and Veterinary Medicine, Bucharest, Romania; 5 Department of Veterinary Clinical Sciences, City University of Hong Kong, Hong Kong, China

**Keywords:** Heartworm disease, Migration, Microfilaria, Urine

## Abstract

*Dirofilaria immitis* is a mosquito-borne nematode-causing canine heartworm disease, with adult worms localized in the pulmonary arteries and right heart. In rare cases, ectopic migration might occur, and adults and blood circulating microfilariae can be found in unusual organs or fluids (e.g., eyes, abdominal cavity, bone marrow, and urine). A 17-year-old mixed-breed female dog was presented in a private veterinary clinic in Italy for hematuria and dysuria. Physical examination showed cardiac mitral murmur with marked respiratory distress and cyanotic mucous membranes after handling. Abdominal ultrasounds revealed a non-specific chronic cystopathy, while the echocardiography showed enlargement of the right heart associated with tricuspid insufficiency and mitral regurgitation, with the presence of an adult filariae in the right ventricular chamber. Circulating microfilariae were observed in the blood smear and molecularly identified as *D. immitis*. Unusual microfilaruria was detected in the urine sediment. Data presented raise awareness about the occurrence of microfilariae in unusual locations, such as the bladder, suggesting the need of a thorough clinical and laboratory assessment where *D. immitis* is endemic.

## Introduction

*Dirofilaria immitis* (Onchocercidae, Dirofilaridae) is a mosquito-borne filarial nematode, responsible for heartworm diseases (HWD) in dogs worldwide (Nelson et al. 2005). The infection occurs when third-stage larvae (L3) are released by the intermediate hosts (i.e., mosquitoes of the genera *Aedes*, *Anopheles*, and *Culex*) on the wounded skin of the definitive host, soon after the blood intake (Dantas-Torres and Otranto 2013). The L3 migrate from the subcutaneous or sub-serosal tissues of the host undergoing molting to L5 immature (i.e., 50–70 days), eventually reaching pulmonary arteries and the right heart in about 5 months (Nelson et al. 2005). Erratic migrations of immature or mature adults might happen, both inside nodules around the eye (Eberhard et al. 1977; Goh et al. 2023) or in the anterior chamber (Dantas-Torres et al. 2009; Hayasaki et al. 2013). Immature or adult helminths were also found in the abdominal cavity and scrotum (i.e., during elective neutering) (Kang et al. 2011; Kayama et al. 2018), brain (Hamir 1987) and also as multifocal ulcerative subcutaneous nodules (Goh et al. 2023; Silva et al. 2023). Once the adults reach sexual maturity and copulate, microfilariae are released and found circulating in the blood (Nelson et al. 2005), being rarely retrieved in bone marrow (Lensi et al. 2023), bile ducts (Sevimli et al. 2007) and urine (Kaewthamasorn et al. 2008). These unusual findings may represent a diagnostic challenge as practitioners do not expect such erratic microfilariae. Therefore we described an unusual case of microfilariae by *D. immitis* detected during urinalysis of a dog referred for hematuria.

### Results and discussion

**Fig. 1:**
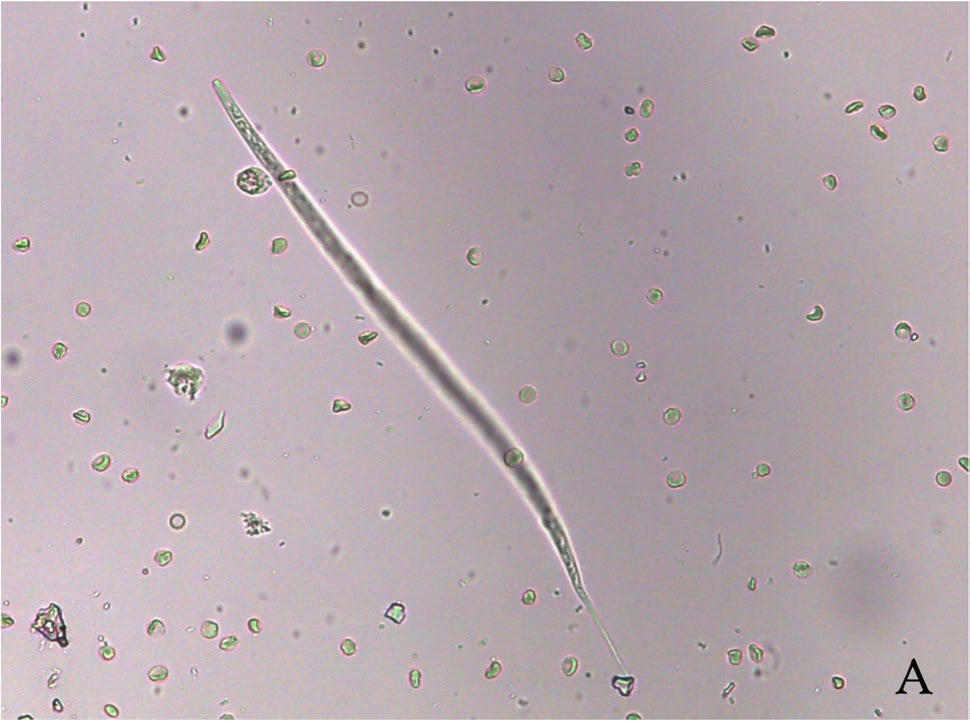
Photomicrograph of urine sediment of a dog showing microfilaria of *Dirofilaria immitis*

**Fig. 2:**
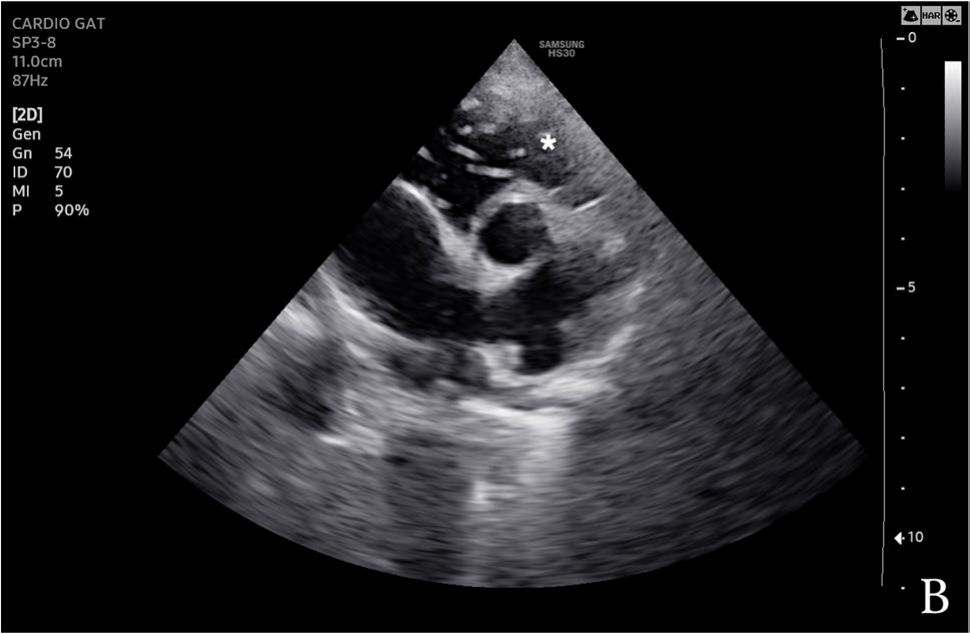
Echography image of a dog’s heart infected with *Dirofilaria immitis*, with the presence of echogenic lines in the right ventricular chamber (*)

A 17-year-old mixed-breed female dog was presented in a private clinic at Brindisi, Italy, with an acute onset of hematuria and dysuria, as principal clinical signs. History reported that the dog lived in an endemic area for *D. immitis* (Mendoza-Roldan et al. 2020) nearby the seaside and received external acaricides treatments (4 ml of spot solution containing 400 mg of Imidacloprid plus 2500 mg of Permethrin) only in the summer period. Physical examination was unremarkable apart from the presence of both right- and left-sided heart murmurs with marked dyspnoea and cyanotic mucous membranes after handling. Echocardiography revealed enlargement of the right heart chambers and pulmonary artery, septal and right ventricle wall thickness, and tricuspid regurgitation (143.78 cm/s). The aortic and pulmonary flowmetry was within normal limits. Adult filariae were present in the right ventricular chamber (Fig. 1). The left ventricle wall was within normal thickness, though increased ventricular function indices (i.e., sphericity index <  1.6 and mitral regurgitation; 132.76 cm/s) were detected. Echocardiographic findings were typical for right heart failure associated with mitralic insufficiency and heart remodeling. Abdominal ultrasounds showed a poorly filled bladder, with an apparently thickened wall and endoluminal irregular surface, with anechoic contents and hyperechoic echoes in suspension; the above suggested for a chronic cystopathy. The presence of a rounded and anechoic lesion (i.e., 2.69 mm in diameter), at the level of the cortex of the left kidney was also detected. Furthermore, moderate abdominal effusion was observed, but the owner denied the sampling. The complete blood cell count revealed a low-grade, non-regenerative anemia, marked presence of rouleaux, moderate neutrophilia (10,200 (3500 − 9300)) (segmented neutrophils), eosinophilia and, moreover, microfilariae were visualized at the blood smear. Serum biochemical analysis revealed increased value for C reactive-protein (4.52 (0.00 − 1.00)) and ferritin (449 (80 − 270)). Blood and biochemical findings suggested a non-specific inflammatory/infective disease and the serum sample resulted positive for *D. immitis* antigen using a commercial Enzyme Linked Immunosorbent Assay (ELISA Novatec® kit for CanL and Dirocheck Zoetis® for HW). Therefore, blood was submitted to DNA extraction using GenUP™ Blood DNA Kit according to the manufacturer’s instructions and to real-time PCR (qPCR) for discrimination of *Dirofilaria* spp. using melting curve analysis (Latrofa et al. 2012), resulting positive for *D. immitis* (melting temperature of 75.5°C). The urine sample collected by spontaneous urination was turbid, with the presence of blood (hematuria). Urine analysis revealed epithelial cells (i.e., squamous and transition cells in small aggregates), high protein quantity, high UPC (urine protein/creatinine) ratio (1.74 (< 0.5)) and presence of some microfilariae in the sediment (Fig. 2). Urine analysis findings were suggestive for inflammatory/infectious lower urinary tract disease, but the owner refused to perform the cystocentesis.

Due to the high risk of thromboembolism, the dog underwent treatment with ivermectin (12 mcg/kg orally every 14 days during 6 months) for a gradual decline of microfilariae, prednisolone (0.5 mg/kg/SID, for 3 days) to reduce the potential adverse reaction in this highly microfilaremic dog associated with doxycycline (10 mg/kg orally for 30 days) which targets the bacterial endosymbiont *Wolbachia* (Manoj et al. 2021). Furthermore, the treatment was associated with benazepril (0.25 mg/kg BID orally during 6 months) and furosemide (1 mg/kg/BID orally during 6 months) to support heart failure. After treatment, the dog fully recovered, but died suddenly after 5 months, being the necropsy not authorized by the owner.

We report an unusual microfilaruria by *D. immitis* in an infected dog. Three other microfilaruria cases have been reported in literature, being two associated with cystitis and renal failure (Kaewthamasorn et al. 2008; Monobe et al. 2017), and one without urinary tract disorders (Colak et al. 2020). Microfilaria of *D. immitis* in urine represent an unusual laboratory finding in infected dogs, as it might be a result of the outcomes of the HWD (i.e., glomerulonephritis and renal failure) or even associated with inflammation or hemorrhage of the lower urinary tract, in infected animals (i.e., bacterial cystitis, neoplasia) (Osborne et al. 1995). Moreover, microfilariae may occlude and cause the rupture of small vessels with their release in the lower urinary tract (Venco et al. 2005). In addition, the immune complex and fibrin deposition in the glomeruli may lead to glomerulonephritis and renal failure, also in association with the presence of microfilaria in the urine (Venco et al. 2005). In the present case, the physical examination was typical of HWD, with respiratory distress and right-sided heart insufficiency, confirmed by echocardiographic findings.

Though the presence of microfilariae at urinalysis is an occasional finding (i.e., probably due to the inflammation and hemorrhage of the lower urinary, and to the passage of red blood cells, epithelial cells, and microfilariae) this occurrence should be considered in the diagnosis of HWD in endemic areas. Indeed, the dog was referred primarily for hematuria and dysuria associated with respiratory clinical signs. This case report raises awareness about the occurrence of microfilariae in unusual locations, such as the bladder, suggesting that a thorough clinical and laboratory assessment should be carried out in endemic areas for *D. immitis*.

## Data Availability

Not applicable.
